# Voltammetric Detection of Urea on an Ag-Modified Zeolite-Expanded Graphite-Epoxy Composite Electrode

**DOI:** 10.3390/s8095806

**Published:** 2008-09-22

**Authors:** Florica Manea, Aniela Pop, Ciprian Radovan, Plamen Malchev, Adriana Bebeselea, Georgeta Burtica, Stephen Picken, Joop Schoonman

**Affiliations:** 1 “Politehnica” University of Timişoara, P-ta Victoriei, nr.2, 300006, Timişoara, Romania; E-mails: aniela.pop@chim.upt.ro (A.P.); adriana.bebeselea@chim.upt.ro (A.B.); georgeta.burtica@chim.upt.ro (G.B.); 2 West University of Timişoara, Laboratory of Electrochemistry, Str. Pestalozzi nr.16, 300115 Timişoara, Romania; E-Mail: radovan@cbg.uvt.ro (C. R.); 3 Delft University of Technology, 2600 GA Delft, The Netherlands; E-mails: p.g.malchev@tudelft.nl (P.M.); s.j.picken@tudelft.nl (S.P.); j.schoonman@tudelft.nl (J.S.)

**Keywords:** Urea determination, expanded graphite-Ag-zeolite-epoxy composite electrode, electrocatalytic effect

## Abstract

In this paper, a modified expanded graphite composite electrode based on natural zeolitic volcanic tuff modified with silver (EG-Ag-Z-Epoxy) was developed. Cyclic voltammetry measurements revealed a reasonably fast electron transfer and a good stability of the electrode in 0.1 M NaOH supporting electrolyte. This modified electrode exhibited moderate electrocatalytic effect towards urea oxidation, allowing its determination in aqueous solution. The linear dependence of the current versus urea concentration was reached using square-wave voltammetry in the concentrations range of urea between 0.2 to 1.4 mM, with a relatively low limit of detection of 0.05 mM. A moderate enhancement of electroanalytical sensitivity for the determination of urea at EG-Ag-Z-Epoxy electrode was reached by applying a chemical preconcentration step prior to voltammetric/amperometric quantification.

## 1. Introduction

The determination of urea is essential for a number of applications, including clinical diagnosis, food processing, agricultural process and environmental protection [1-5]. Because of very fast and high expansion of industrial and farming activity, the presence of urea in water is increasing. Also, high quantities of urea are present in municipal wastewaters because about 80% the nitrogen in fresh urine is bound in urea [6].

Several analytical procedures have been developed for the analysis of urea in aqueous samples. Most of these investigations on the determination of urea have been based on measuring the changes of ammonia enzymatically released during the hydrolysis of urea. There are several analytical procedure based on the optical methods [7, 8] and electrochemical techniques. In general, the assay of urea by electrochemical techniques means the use of enzyme-modified electrodes by potentiometric and amperometric methods [1, 3, 4]. Though, a non-enzimatic amperometric detection of urea in aqueous solution at poly(Ni-cyclam) film-modified glassy carbon electrode was reported by Ferrer [2].

Zeolite-modified electrodes (ZMEs) have been drawing attention as chemically modified electrodes (CMEs) because of synergistic combination of zeolite features with electrochemical interfaces [9-12]. ZME can be exploited as electrochemical sensors in relation with zeolite's properties, e.g., ion-exchange capacity, molecular selectivity, and catalyst-assisted reactivity. Moreover, metal ion-doped zeolites allow exploitation of ion-exchange capacity of zeolite for the development of electrochemical sensors for the sensing non-electroactive inorganic or organic species [10, 13]. Also, the zeolite-supported electrocatalyst can be exploited to improve the performance of analytical sensing device. For the electrochemical detemination of urea, the clinoptilolite has been exploited for the preparation of biosensors by covalent binding of enzymes to the surface of an ion-sensitive membrane made up of a zeolite-polymer matrix [14]. In this situation, the electrochemical strategy was based on the clinoptilolite affinity for ammonium to determine urea using urease. The main disadvantages of the ammonium sensor were its long response time and the limited stability of the urease layer.

In the present study, an expanded graphite-Ag-modified zeolite-epoxy composite (EG-Ag-Z-Epoxy) electrode was prepared from Romanian natural zeolitic volcanic tuff modified with silver, and electrochemically characterized for urea detection.The catalytic electrochemical oxidation of urea on (EG-Ag-Z-Epoxy) electrode was investigated. The kinetic parametrs for the heterogeneous electron transfer at EG-Ag-Z-Epoxy electrode (the rate constant and the transfer coefficient) were estimated using Laviron's treatment [15]. The electrode was examined for its electrocatalytic activity toward urea oxidation by cyclic voltammetry (CV), liner-scan voltammetry (LSV), differential-pulse voltammetry (DPV), square-wave voltammetry (SWV), and chronoamprometry (CA).

## 2. Results and Discussion

The prepared EG-Ag-Z-Epoxy composite electrode showed good mechanical strength and electrical conductivity (18.28 S·cm^−1^).

### 2.1. Surface characterization

**Figure 1.**
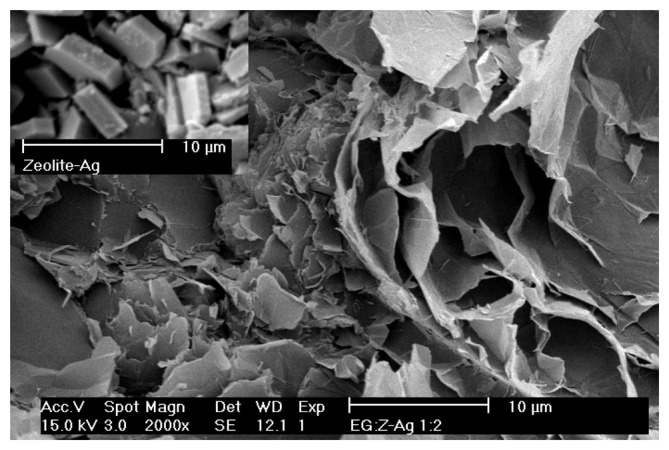
SEM image of EG-Ag-Z-Epoxy composite electrode. Inset: SEM image of Ag modified zeolite.

SEM imaging has been used to provide qualitative information about distribution of expanded graphite and Ag-doped zeolite zones and some surface features of this composite electrode. Figure 1 illustrates comparatively the images of Ag-zeolite particles and EG-Ag-Z-Epoxy composite electrode and exhibited a closely spaced expanded graphite zones with random distribution and orientation due to the irregular shapes of both the expanded graphite particles and Ag-zeolite particles in epoxy matrix. The Ag-zeolite particles are invisible on the composite surface because they are completely embedded into the epoxy resin, and are rarely exposed at the electrode surface. In addition, a SEM image of Romanian Ag-doped zeolite is shown in the inset of Figure 1 for comparison.

### 2.2. Voltammetric measurements

**Figure 2.**
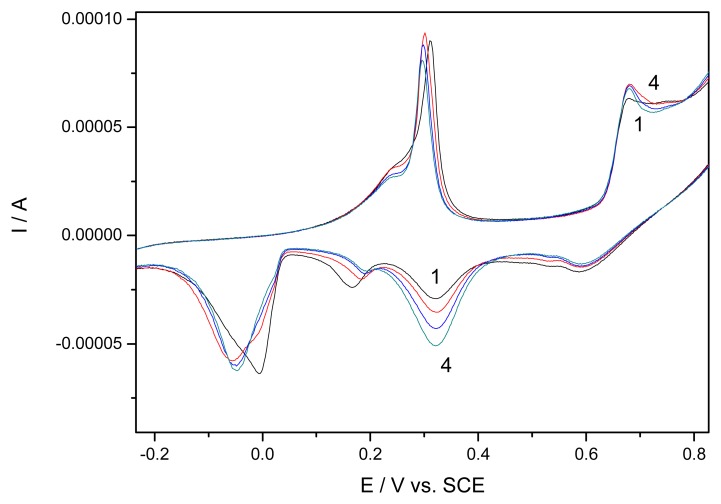
Detail of repetitive cyclic voltammograms (CVs) of EG-Ag-Z-composite electrode in 0.1 M NaOH supporting electrolyte; potential range of −0.5 to 1.25 V/SCE; scan rate 0.05 Vs^−1^; 1-4: initial CV-the fourth repetition of CV

Figure 2 depicts a detail of CVs of EG-Ag-Z-Epoxy composite electrode in 0.1 M NaOH supporting electrolyte involving repetitive first scans. It can be seen that the EG composite electrode containing Romanian zeolite with small amount of Ag^+^ cations distributed randomly in the zeolite structure displays complex behaviour with several electrochemically distinct species. The anodic part of the voltammogram is characterized by the occurrence of the anodic peaks corresponding to the electroformation of soluble [Ag(OH)_2_]^−^ complex species (the first peak, shoulder), to the electroformation of Ag_2_O (the second peak), and to the formation of AgO (the third peak) [16,17]. The cathodic part of the cyclic voltammograms is characterized by the occurrence of cathodic peaks corresponding to the electroreduction of AgO to Ag_2_O, the reduction of Ag^+^ located in different sites of zeolite [12]. However, it must be noticed that the anodic current is very stable, while the catodic current is stable up to the reduction of Ag^+^ species. The stability of CVs could be owned to time-consuming process of the soakening of embedded zeolite particles.

**Figure 3.**
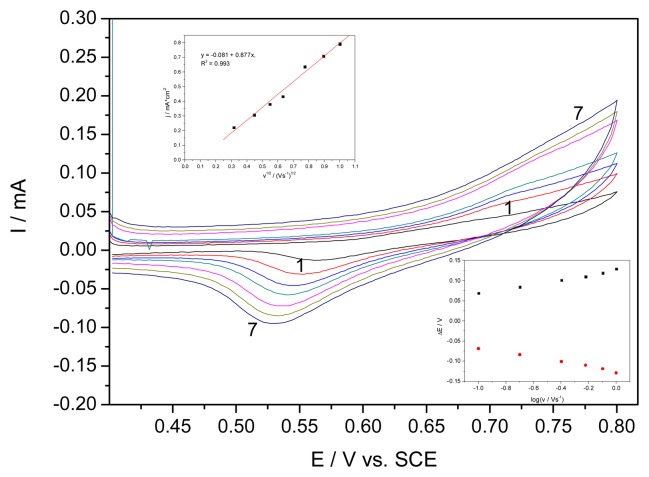
Cyclic voltammograms (CVs) of EG-Ag-Z-composite electrode in 0.1 M NaOH supporting electrolyte at different scan rates: 1-0.01, 2-0.02, 3-0.03, 4-0.04, 5-0.06, 6-0.08, 7-0.1 V·s^−1^; Insets: a)-The anodic peak current versus the square root of scan rate, b)-Experimental variation of ΔE versus the logarithm of the scan rate.

An investigation of the effect of the scan rate on the peaks currents and potentials corresponding to the redox AgO/Ag_2_O couple was carried out (Figure 3). The linear proportionality of the current peak vs. square root of the scan rate (Inset a of Figure 3) indicated that the process was generally diffusion step controlled and the practically zero intercepts suggested that adsorption steps and surface interactions were negligible. Also, from the variation of peak potentials with scan rate (Inset b of Figure 3), using the treatment proposed by Laviron [15], kinetics parameters for the heterogeneous electron transfer at EG-Ag-Z-Epoxy composite electrode were determined in relation with redox couple of Ag(II) and Ag(I) oxides. Taking into account that for scan rates higher than 300 mVs^−1^, the difference between the anodic and cathodic peak was higher than 200 mV, and the values of ΔE = Ep-E°' were proportional to the logarithm of scan rate, the transfer coefficient (α) and the apparent charge transfer rate constant (k_s_) for electron transfer between the electrode and surface deposited layer were determined. A plot of Ep=f(logv) yields two straight lines with slope equal to 2.3RT/αnF and 2.3 RT/(1-α)nF for the cathodic and anodic peak. Using such a plot and the equation:

(1) $$\log k_{s}=\alpha \log(1-\alpha )+(1-\alpha )\log\alpha -\log(RT/\text{nFv})-\alpha (1-\alpha )nF\Delta Ep/2.3RT$$

where the values of α and k_s_were 0.42 and 0.36 s^−1^, respectively, for EG-Z-Ag-Epoxy in the presence of 0.1 M NaOH subjected to AgO /Ag_2_O couple.

**Figure 4.**
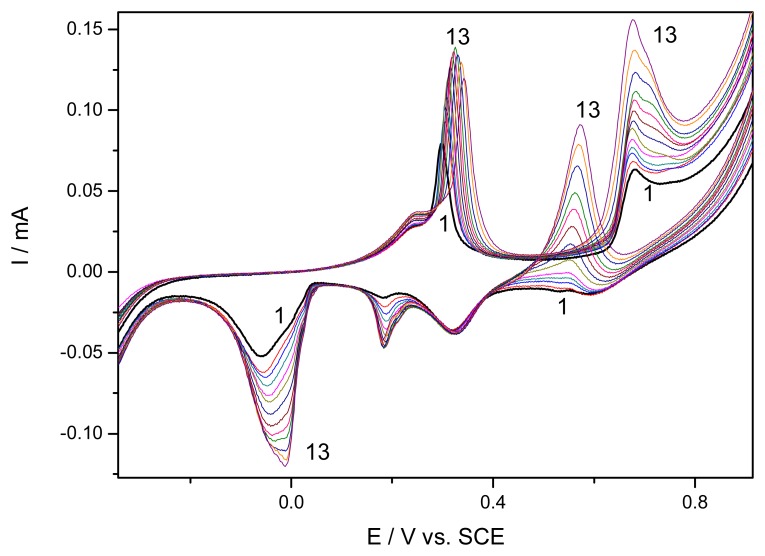
Cyclic voltammograms of EG-Ag-Z-Epoxy composite electrode in 0.1 M NaOH supporting electrolyte (1) and in the presence of different urea concentrations: 2-0.1 mM; 3-0.2 mM; 4-0.3 mM; 5-0.4 mM; 6-0.6 mM; 7-0.8 mM; 8-1 mM; 9-1.2 mM; 10-1.4 mM; 11-1.8 mM; 12-2.2 mM; 13-2.6 mM; potential scan rate 0.05 Vs^−1^; potential range: −0.5 to 1.25 V

A series of resultant CVs obtained directly over the concentration range 0.1 mM – 2.6 mM for urea standard solutions, corresponding to curves 1-13, is illustrated in Figure 4. The anodic current peaks corresponding to AgO formation increased progressively linearly with urea concentration. Also, the two cathodic current peaks recorded at about 0 and 0.2 V vs. SCE increased with urea concentrations but the current within this potential range was not considered due to the fact it was not stable in the absence of urea (see Figure 2). A very unexpected and unusual oxidation peak occurred immediately after the reduction of AgO to Ag_2_O on backward scanning in cathodic sense, which increased also linearly with urea concentration.

**Figure 5.**
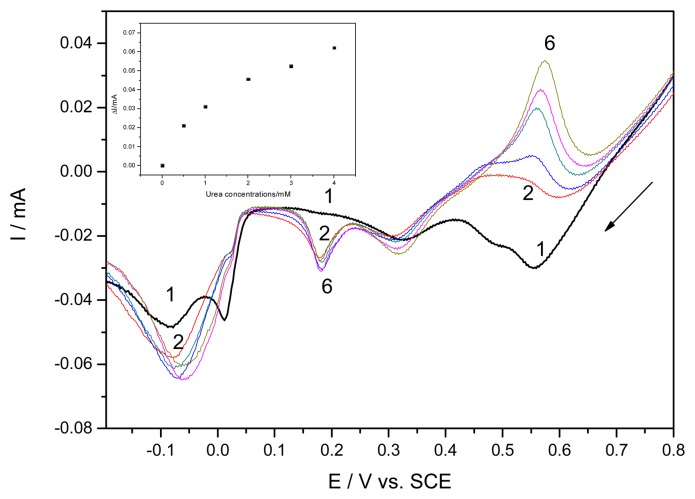
Linear scan voltammograms of EG-Ag-Z-Epoxy composite electrode in 0.1 M NaOH supporting electrolyte (1) and in the presence of different urea concentrations:2-0.5 mM; 3-1 mM; 4-2 mM; 5-3 mM; 6-4 mM; potential scan rate 0.05 Vs^−1^; potential range:+1.25 to−0.5 V (arrow indicates the scanning direction). Inset: The dependence of linear scan voltammetric signal recorded at +0.55 V/SCE vs. urea concentrations.

**Figure 6.**
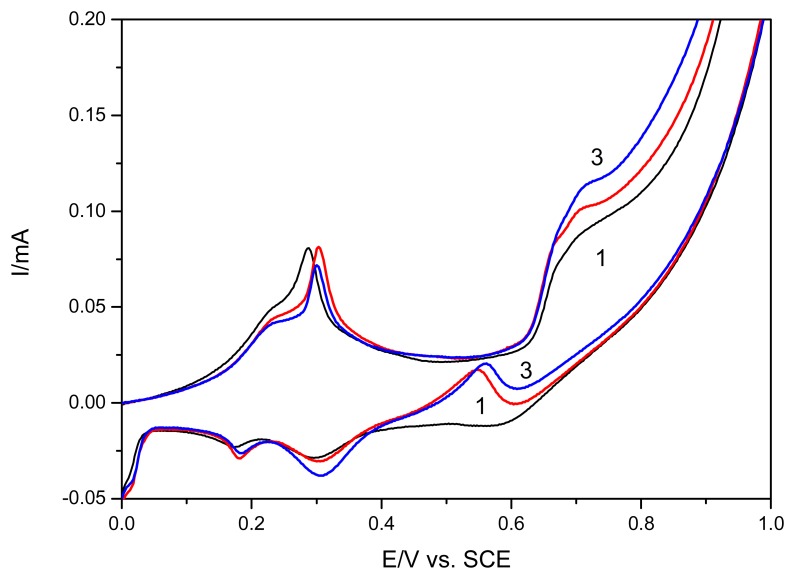
Cyclic voltammograms of EG-Ag-Z-Epoxy composite electrode in 0.1 M NaOH supporting electrolyte (1) in the presence of 0.8 mM urea (2) and in the presence of 0.2 mM ammonium (3).

The anodic current from forward branches of CVs recorded at +0.68 V/SCE depends linearly on the urea concentration in the range 0.1-2.6 mM, with the sensitivity of 0.033 mA·mM^−1^. Also, under the same conditions, the current recorded on backward scan at the potential about +0.55 V vs. SCE showed a better sensitivity, as 0.042 mA·mM^−1^. For both potential values, the linear calibration plots of the current peaks vs. concentration with very good correlation parameter higher than 0.99 were obtained. The linear voltammetric data recorded on both direct and reverse scanning were obtained in the presence of different urea concentrations. As example, in Figure 5 the linear scan voltammograms obtained on reverse scanning (from 1.25 V to −0.5 V vs. SCE) are presented, and a non-linear dependence of the current peak height vs. urea concentration was reached (inset of Figure 5). This unexpected behaviour could be the result of continuously remove of the surface layers of Ag_2_O in the presence of ammonium as a product of catalyzed hydrolysis of urea with formation of soluble complex of diammine silver (Ag(NH_3_)_2_^+^), thus allowing the oxidation to proceed as the electrode is not oxidized in depth [18]. The CVs shown in Figure 6 present the comparative results in the presence of urea and ammonium ions and the behaviour similiarity, as increase of the secondary oxidation peak, support this afirmation.

### 2.3. Electrocatalytic voltammetric/amperometric determination of urea

**Figure 7.**
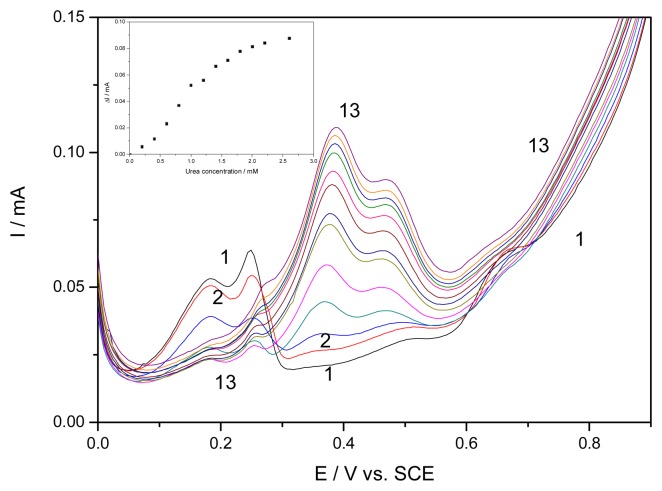
Differential pulse voltammograms recorded on EG-Ag-Z-Epoxy composite electrode with a potential scan rate 0.05 Vs^−1^ between 0 and 1V vs. SCE in a 0.1 M NaOH supporting electrolyte (1) and in the presence of different urea concentrations: 2-13: 0.2 mM -2.4 mM, urea concentration adding step of 0.2 mM. Inset: The dependence of differential pulsed voltammetric signal recorded at +0.38 V/SCE vs. urea concentrations

The results obtained by using the differential pulse voltammetry on EG-Ag-Z-Epoxy composite electrode in 0.1 M NaOH and in the presence of different urea concentrations, and the variation of pulsed voltammetric peaks with urea concentrations (Inset of Figure 7) are shown in Figure 7. The electroanalytical parameters for the concentration ranges where linear proportionality was reached are also presented in Table 1. It has to be mentioned that analytical singnals were recorded after preliminary conditioning at the potential value of +1.25V/SCE for 30 seconds in the presence of urea. No analytical signals occurred without preconditioning, an aspect that strengthens the idea that the signal corresponds to a soluble complex of diammine silver.

Differential pulsed voltammetric experiments show that the peaks corresponding to the electroformation of soluble [Ag(OH)_2_]^−^ complex species and the electroformation of Ag_2_O are influenced by increasing urea concentration. This may be the result of the competitive adsorption between ammonium and hydroxyl ion or a change in mechanism. The both peaks results from the formation of a soluble complex between ammonium and silver species.

**Figure 8.**
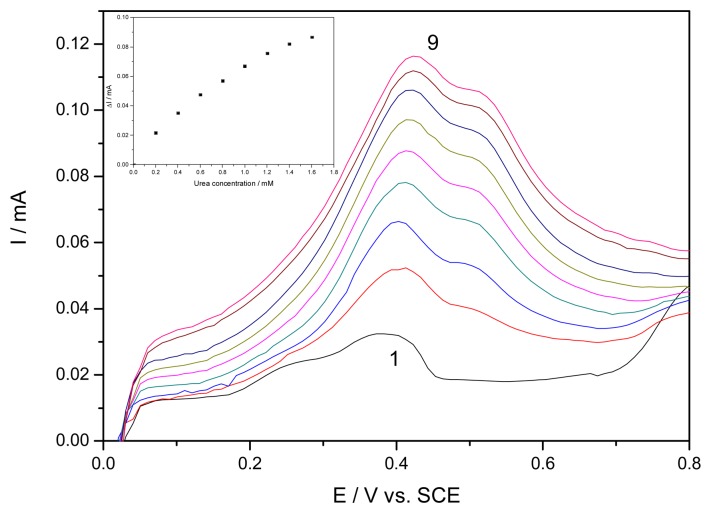
Square-wave voltammograms recorded on EG-Z-Ag-Epoxy electrode with a potential scan rate 0.05 Vs^−1^ between 0 and 1V vs. SCE in a 0.1 M NaOH supporting electrolyte (1) and in the presence of different urea concentrations: 2-9: 0.2 mM −1.6 mM, urea concentration adding step of 0.2 mM. Inset: The dependence of square-wave voltammetric signal recorded at + 0.41 V/SCE vs. urea concentrations.

The similar results were obtained also by using and square-wave voltammetry, and the voltammograms in relation with urea concentrations are presented in Figure 8. For the whole concentration range studied no linear increase of the amperometric signal vs. concentration was reached (inset of Figure 8). However, it has to be mentioned that shape of square-wave voltammograms is different versus differential-pulsed voltammograms, and the presumptive electroformation of soluble [Ag(OH)_2_]^−^ complex species was not evidenced.

**Figure 9.**
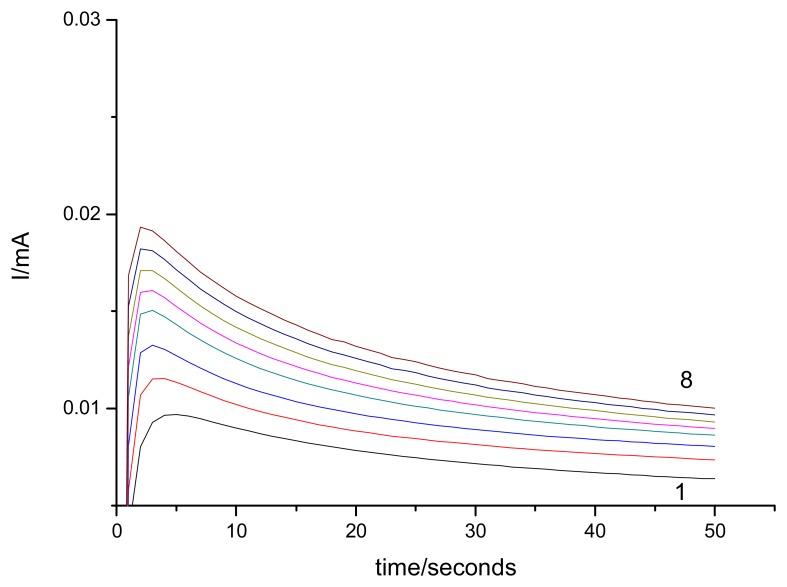
Chronoamprometric response of EG-Ag-Z-Epoxy composite electrode recorded at +0.55 V/SCE to successive adding of 0.4 mM urea (1-0.1 M NaOH supporting electrolyte; 2-8: 0.4 mM-2.8 mM urea).

Batch amperometric measurements at constant applied potential (+0.55 V vs. SCE) prove that EG-Ag-Z-Epoxy composite electrode works well as amperometric sensor for urea determination. A typical example of amperometic response to successive adding of 0.4 mM urea is presented in Figure 9.

**Figure 10.**
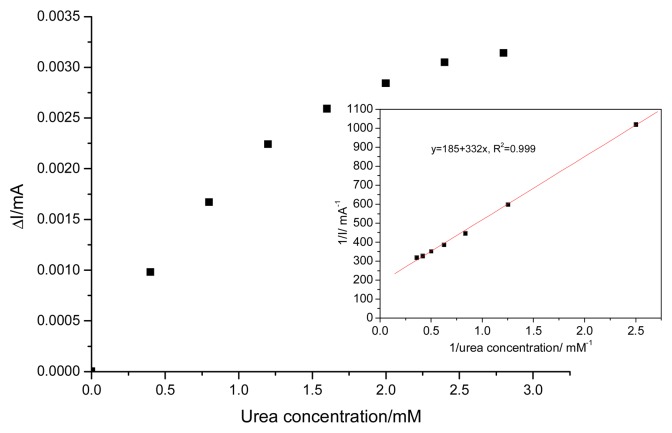
Calibration plot of EG-Z-Ag-Epoxy electrode for successive adding of 0.4 mM urea in 0.1 M NaOH; applied potential +0.55 V vs. SCE, conditioning at potential value of +1.25V vs. SCE for 30 seconds; Inset: Lineweaver Burke plot.

When the concentration of urea was higher than 1.4 mM, a response platform as a “catalytic saturation” was observed, showing a characteristic in a formal accordance with the Michaelis-Menten catalytic kinetic mechanism. The kinetic parameters could be obtained from the electrochemical version of the Lineweaver-Burk equation [19]. The apparent Michelis-Menten type constant, *K*′*_M_*, and *I_max_* were determined by analysis of the slope and intercept of the plot of the reciprocals of the steady-state current recorded at 50 seconds versus urea concentration (Figure 10). The *K*’*_M_* value of the EG-Ag-Z-Epoxy electrode was 1.6 mM and *I_max_* =0.05 mA. Also, the sensitivity calculated as *I_max_*/*K*’*_M_* ratio was 0.03 mA·mM^−1^.

Under these working conditions, electroanalytical performances of EG-Z-Ag-Epoxy for urea determination was reached. Based on sorption properties of clinoptilolite for urea [20], the further experiments were carried out to apply a preconcentration-voltammetric detection scheme to get the better electroanalytical performances for urea detection.

### 2.4. Influence of accumulation time

The accumulation time is very important for using EG-Z-Ag-Epoxy electrode in a preconcetration-voltammetric detection scheme, because it could influence the degree of adsorption on the electrode surface. An important role that may be played by zeolite is its availability to concentrate species within its porous structure [21]. This would also increase the amount of species available for reaction. To determine the enhancement factor as the ratio of peak current after and before sorption process, the effect of accumulation time on anodic peak current densities were investigated by DPV. Figure 13 shows the dependence of the useful signal corresponding to the anodic peak current corresponding to urea determination and enhancement factor on the accumulation time for 0.4 mM urea. By increasing accumulation time up to 15 minutes the amount of urea at electrode surface increased leading to the enhancement of useful signal. The enhancement factor of about 3 revealed an effective concentration effect of EG-Z-Ag-Epoxy electrode on urea. Also, it is well-known that the kinetics of the accumulation process depends on the nature of zeolite-modified electrode. The accumulation time of 15 minutes needed to reach the equilibrium suggested a diffusion effect of the solution in the bulk of the electrode, may be due to the electrode surface porosity [10]. The better electroanalytical performance obtained by using preconcetration- differential pulsed voltammetric detection scheme is gathered also in Table 1.

The equations of the linear dependencies of the current vs. urea concentration, with corresponding characteristic analytical parameters, working conditions and techniques applied in various situations are gathered in Table 1. The limit of detection (LD) determined as 0.05 mM urea, was evaluated based on 3S_B_/b [22], where S_B_ is the standard deviation of the mean value of 5 voltammograms of the blank and b is the slope of the straight line in the analytical curve by using square-wave voltammetry. The reproducibility of the electrode using the above-mentioned technique was evaluated for three replicates measurements of urea detection. The relative standard deviation (RSD) of 3.2 % showed the good reproducibility of the electrode. A recovery test was also performed by analyzing three parallel tap water samples, which contain 60.07 mg·dm^−3^ urea. This test was run in 0.1 M NaOH as supporting electrolyte and a recovery of 98 % was found.

## 3. Experimental Section

The expanded graphite-Ag-zeolite-epoxy (EG-Z-Ag-Epoxy) composite electrode was obtained from two-component epoxy resin (LY5052, Araldite) mixed with conductive expanded graphite (EG) fillers powder (Conductograph, SGL Carbon) and silver modified zeolite (clinoptilolite). The ratio between the components was chosen to reach 20 weight percent (w/w) content of expanded graphite, 40 weight percent (w/w) content of silver-modified zeolite. Ag-modified zeolite with a content of 0.008 mg Ag /g zeolite was prepared using natural zeolite from Mirsid, Romania, with 68% wt. clinoptilolite as we previously described [23]. It was not possible to add the full amount of EG and Z-Ag to the matrix resin directly, due to the high surface area of the graphite flakes, therefore, the mixing was performed in a roll-mill at room temperature. The two parts of the epoxy were mixed together and the full amount of the EG was added in steps forming a thick paste. Then the epoxy was treated in a hot press at 50 °C for 60 minutes. Simultaneously the material was shaped in a plate of 1 mm thickness. The plate was slowly cooled down (for about 12 h) to the room temperature. The electrical conductivity of the electrode by four points resistance measurement [24] was found to be 18.28 S·cm^−1^. Discs with a surface area of 19.63 mm^2^ were embedded in polyethylene and electrical contacts were made using a copper wire. Prior to use, the working electrode was gradually cleaned, first polished with abrasive paper, then polished with (0.3-0.1 µm) alumina powder in aqueous suspension on a pad and rinsing with distilled water. A Scanning Electron Microscope (Philips CM30T) was used to observe the working electrode surface.

The electrochemical behaviour/performance of the electrode was studied by cyclic voltammetry (CV), linear-scan voltammetry (LSV), square-wave voltammetry (SWV), differential pulse voltammetry (DPV) and chronoamperometry (CA). Subsequently, an electrochemical pre-treatment by three repetitive cyclings between −0.5 V to 1.25 V vs. SCE in 0.1 M NaOH supporting electrolyte was performed. All measurements were carried out using an Autolab potentiostat/galvanostat PGSTAT 302 (Eco Chemie, The Netherlands) controlled with GPES 4.9 software and a three-electrode cell, with a saturated calomel electrode as reference electrode, a platinum counter electrode and the composite working electrode. DPV is a pulse technique, dependent on applied parameters, i.e., a scan rate of 25 mV·s^−1^, a pulse modification of 50 mV in amplitude and 50 ms in duration at intervals of 200 ms. Also, SWV is a pulse technique and the parameters applied was the frequency of 10 Hz, amplitude of 0.45 mV, and the scan rate of 50 mV·s^−1^.

Accumulation was performed at open circuit potential (OCP) in the analyte solution, the electrode was then removed and rinsed with distilled water, and immersed in the detection cell for recording the voltammetric curve. Regeneration was achieved by mechanical polishing and above-mentioned electrochemical pretreatment.

## 4. Conclusions

An EG-Ag-Z-Epoxy composite electrode prepared from Romanian natural zeolitic volcanic tuff showed good mechanical strength and electrical conductivity. A random distribution and orientation of expanded graphite particles and Ag-zeolite particles in epoxy matrix was shown by SEM.

This composite electrode displayed a complex behaviour in 0.1 M NaOH supporting electrolyte with several electrochemically distinct species, involving the redox couple of AgO and Ag_2_O. The moderate apparent charge transfer rate across the modified electrode interface within the potential range of two types of silver oxides should be correlated with particular structural characteristics of Ag-modified zeolite and suggests a charge transfer mechanism partially based on surface-mediated electron transfer [13].

This modified electrode exhibited an electrocatalytic effect via redox couple of AgO and Ag_2_O towards urea oxidation allowing its determination in aqueous solution. The results obtained by all techniques used, *i.e.*, CV, LSV, DPV, SWV, CA supposed that the mechanism of urea determination by EG-Ag-Z-Epoxy composite electrode in 0.1 M NaOH was the result of continuously remove of the surface layers of Ag_2_O in the presence of ammonium as a product of catalyzed hydrolysis of urea with formation of soluble complex of diammine silver (Ag(NH_3_)_2_^+^).

The electronalytical sensitivity for the determination of urea at EG-Ag-Z-Epoxy composite electrode ranged from 0.030 to 0.065 mA·mM^−1^ function of the technique used.

A moderate enhancement of electroanalytical sensitivity for the determination of urea at EG-Ag-Z-Epoxy composite electrode was reached by applying a chemical preconcentration step prior to voltammetric/amperometric quantification.

Taking into account that the application of this sensor in the electroanalysis of real water/wastewater samples or clinical diagnosis, our future research will be focused on to evaluate the influence of urea-associated chemicals as potential interfering agents.

## Figures and Tables

**Figure 11.**
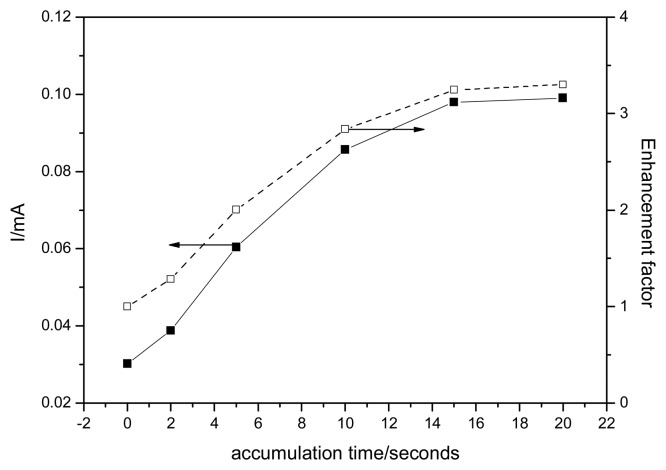
Peak current density responses and enhancement factor for the oxidation of 0.4 mM urea for EG-Z-Ag-Epoxy electrode, as a function of the accumulation time, with background current density subtraction. Detection was performed in 0.1 M NaOH supporting electrolyte by DPVs recorded at 0.38 V/SCE.

**Table 1.** Electroanalytical performance of EG-Z-Ag-Epoxy electrode for the detection of urea for the concentration range of 0.2 to 1.4 mM.

**Peak potential**	**Technique used**	**Sensitivity (mA·mM^−1^)**	**Correlation coefficient (R^2^)**
+0.68	CV	0.033	0.994
+0.55*	CV	0.044	0.993
+0.41	SWV	0.058	0.990
+0.38	DPV	0.044	0.991
+0.55	CA	0.030	0.999
+0.38	Preconcentration/DPV	0.065	0.990

* recorded on the reverse scanning of CV
